# Audit of depot antipsychotic side-effect monitoring within a Community Treatment Team (CTT) in the North East of England in 2022

**DOI:** 10.1192/j.eurpsy.2025.1619

**Published:** 2025-08-26

**Authors:** O. A. S. Mohamed, N. Ahuja, S. Siddiqui, S. Aluru, N. Keidan, F. Eissa, J. Taylor

**Affiliations:** 1 Psychiatry, Cumbria, Northumberland, Tyne and Wear Trust, Newcastle upon Tyne; 2 Psychiatry, Cumbria, Northumberland, Tyne and Wear Trust, North Tyneside, United Kingdom

## Abstract

**Introduction:**

This audit was undertaken to look at side-effect monitoring of patients on depot antipsychotics within the North Tyneside CTT. This was the fourth reaudit of this patient group, with the most recent one having been in 2019, highlighting a gap in re-audits during the height of the COVID-19 pandemic.

**Objectives:**

The objective of this audit was to ensure adherence to the following standards, as per National Institute for Health and Care Excellence (NICE) guidelines and local trust guidelines pertaining to antipsychotic monitoring: 1. 100% of patients on depot anti-psychotics should have side effect monitoring, in the form of a Glasgow Antipsychotic Side-Effect Scale (GASS) form, completed every year. 2. 100% of patients on depot anti-psychotics should have a GASS form completed ever, since starting their antipsychotic medication. 3. Completed GASS forms should be accessible on RiO (online noting system).

**Methods:**

Data collection occurred and concluded in March 2022. This involved identifying patients on depot antipsychotics within the CTT, pseudo anonymising them by patient number and basic demographics, then utilising RiO to identify whether they have had antipsychotic monitoring using a GASS questionnaire as per the guidelines outlined above. This data was then represented in excel form, allowing us to appraise adherence to guidelines and changes from previous audits performed on this topic. We then utilised a traffic light system where green represented a score of 90-100% (compliant), yellow 80-89% (partially compliant), and red 0-79% (non-compliant).

**Results:**

53% of patients had a GASS completed in the past year (red). 95% have had a GASS completed ever (green). 98% of GASS were recorded on RiO (green). Demographic and diagnostic data was also gathered and are available in the full body of the report and poster.

**Image 1:**

**Figure:**
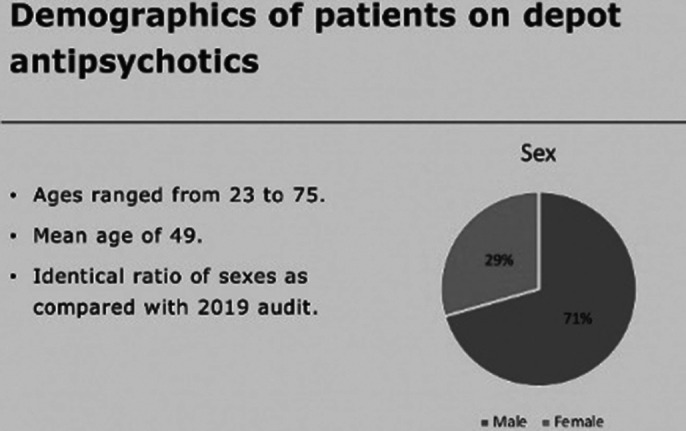

**Image 2:**

**Figure:**
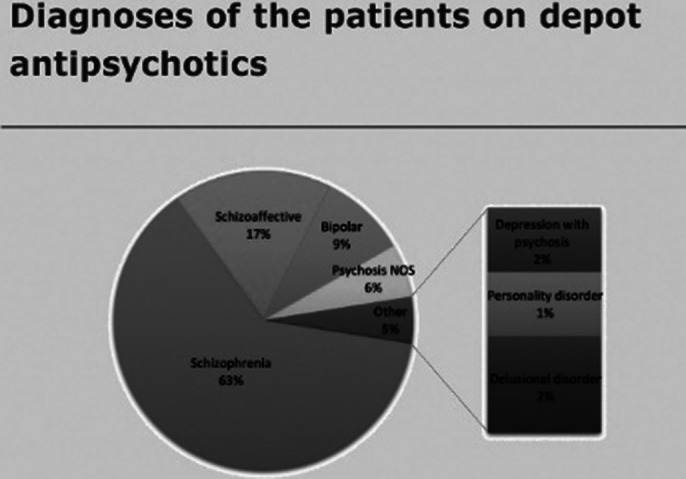

**Image 3:**

**Figure:**
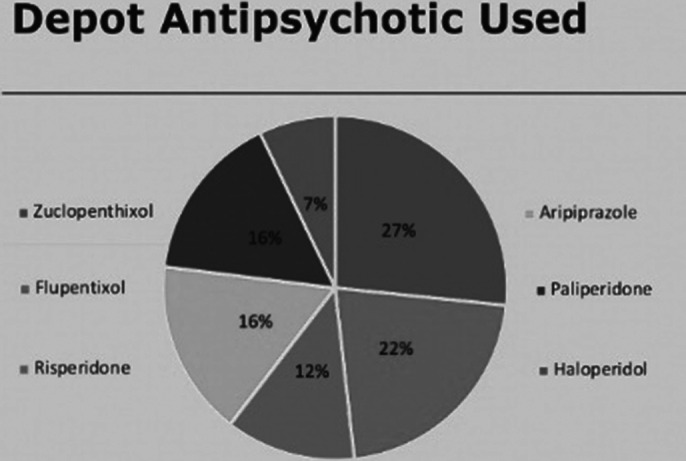

**Conclusions:**

There has been a reduction in the percentage of patients having had a GASS in past year from 88% in 2019 to 53% now. This is likely to represent a disruption to services during the COVID-19 pandemic, forming obstacles to face-to-face reviews of patients. There was an increase in patients on depot antipsychotics from 90 in 2019 to 139 in 2022. There was a marginal improvement in documentation, with 98% of GASS questionnaires recorded on RiO in 2022, compared with 97% in 2019. Following recommendations being made, this will be re-audited in 2024/25.

**Disclosure of Interest:**

None Declared

